# Research on UAV Route Optimization Method Based on Double Target of Confidence and Ambiguity

**DOI:** 10.3389/fnbot.2021.694899

**Published:** 2021-07-14

**Authors:** Huijuan Zhang

**Affiliations:** School of Public Administration, North China University of Water Resources and Hydropower, Zhengzhou, China

**Keywords:** UAV, remote sensing, GIS, path planning, flight planning

## Abstract

In recent years, with the continuous development of drone technology, UAVs are used as unmanned and flightable devices, UAV plays an important role in remote sensing and GIS disciplines. During the flight, no one directly participates in flight-related decisions such as flight routes, path planning, and flight control. In this case, it is necessary to use the computing power of the onboard computer of the UAV system, the computing power of the ground station computer, and related technologies such as detecting sensing, image vision, real-time wireless communication, etc., to develop target planning, decision-making and control algorithms for specific problems, and to solve the problem. Flight planning and flight control issues in machine applications. The UAV route optimization method based on the double target of confidence and ambiguity has positive significance for route optimization and wide application of UAV. In this context, this paper aims to analyze and study the UAV route optimization method based on the two goals of confidence and ambiguity, and optimized the method of drone route. The calculation results show that, compared with other methods, this method can make the UAV not rely on human control, but realize the use of fuzzy control method to identify the target and track the moving target.

## Introduction

With the development and maturity of drones and related technologies, drones are increasingly used in various fields such as defense military, land and resources exploration, forestry security, traffic command and dispatch, etc., especially in the military field. Attention (Messous et al., 2019). As a kind of unmanned and flightable equipment, no one is directly involved in flight-related decisions such as flight routes, flight paths and flight control during flight. It is necessary to use the UAV system onboard computer and ground. The computing power of the station computer, as well as related technologies such as sensing, image vision, real-time wireless communication, etc., develop target-oriented planning, decision-making and control algorithms to solve flight planning and flight control problems in UAV applications. UAV route planning (Zhao et al., 2019) has become the primary problem that must be solved when UAVs are applied to specific fields, and has become one of the hot issues in academic research. The so-called “between strategizing and winning thousands of miles away” plans the flight routes for the drones in advance, and the effect of coordinating and planning the execution of combat tasks can achieve twice the result with half the effort. In this context, this paper aims to analyze and study the UAV route optimization method based on the two goals of confidence and ambiguity (Zhang S. et al., 2019).

## Related Work

Recently, with the sharp rise of computer computing power, the use of various robots has emerged, especially with the use of electronic factory automation machines, such as wafer cutting and positioning, wafer gold wire splicing machines, or the appearance of wafers after printing. The detectors need to use robots instead of manual automation for precise positioning control. With the expansion of the scope of use of robots, human beings gradually need autonomous robots to provide services to improve their quality of life or leisure, such as home cleaning robots or electronic dogs (Arafat and Moh, 2018; Zhao et al., 2018; Zhu et al., 2018). The former uses CCD to capture images and then plan the path and clear them along the way. Dust and hair confetti, which uses a sensor to measure environmental changes and logically judge with a single-chip pre-input control program to respond and interact with the consumer. Machine vision has also been used more and more widely in the detection of drone buildings.

At present, the application of computer vision methods in the field of drones has been continuously improved. It can process image sequences and videos captured from the environment to generate digital and other decision-making information. In many studies, the research topics mostly refer to the visual positioning of robots, which can make up for the shortcomings of precise positioning systems. Many studies are about the visual positioning of robots, which can make up for the shortcomings of precise positioning systems.

## Platform Introduction

### Equipment Introduction

The earliest use of image processing technology was used to improve the quality of images transmitted by submarine cables between London and New York at the beginning of the century. By the 1970s, Leese, Kantaros and Zavlanos (2017) and Callegaro and Levorato (2018) used statistical grayscale values to calculate satellite imagery of clouds. Then separate the background and objects. The image subtraction method used by Hayajneh et al. (2018) for image tracking is used to predict and track real-time grayscale images using Kalman filter (Alzugaray et al., 2017). The invariant moment method used in this paper is very useful for two-dimensional object recognition and has been used in aircraft identification (Moradi et al., 2018). Paul et al. (2013) proposed a new vision-based target detection and positioning system, and the experimental resulted verify the effectiveness of the framework for autonomous monitoring of drones. Minaeian et al. (2016) discussed that moving objects can be classified according to their shape, motion characteristics or texture. Professor Hu of the University of Shenzhen (Zhang J. et al., 2019), Lyu et al. (2018), proposed Fuzzy Set Theory, which quantifies people's ideas to deal with things that have no explicit values. There are quite a few papers related to drones, mainly for path-following drones and image-guided tracking drones. The main method is to separate the background and objects to track the target.

Because there is only one CCD camera in this experimental drone, there is no way to judge the distance between the target and the drone. Therefore, the ultrasonic sensor is used and set on the nose to judge whether the tracking task is completed or not (Erdelj et al., 2017). For the SRF05 ultrasonic sensor, the basic working principle is to give the ultrasonic wave ranging module SRF05 a trigger signal and then emit the ultrasonic wave. When the ultrasonic wave is reflected to the object and reflected back, the SRF05 outputs an echo signal to the time difference between the trigger signal and the echo signal to determine the distance of the object (Hu et al., 2019).

### System Composition

#### System Platform

Unmanned Aerial Vehicle (UAV) During the mission (Messous et al., 2017; Khuwaja et al., 2018; Ranjan et al., 2018; Yan et al., 2018; Ning et al., 2019), the recycling process is a very important and prone to failure phase. The navigation system needs to be able to precisely control the attitude and trajectory of the aircraft. Realizing the automatic landing of drones is an important part of improving the autonomous control capability of drones.

#### UAV Control System

**Figure 1:**
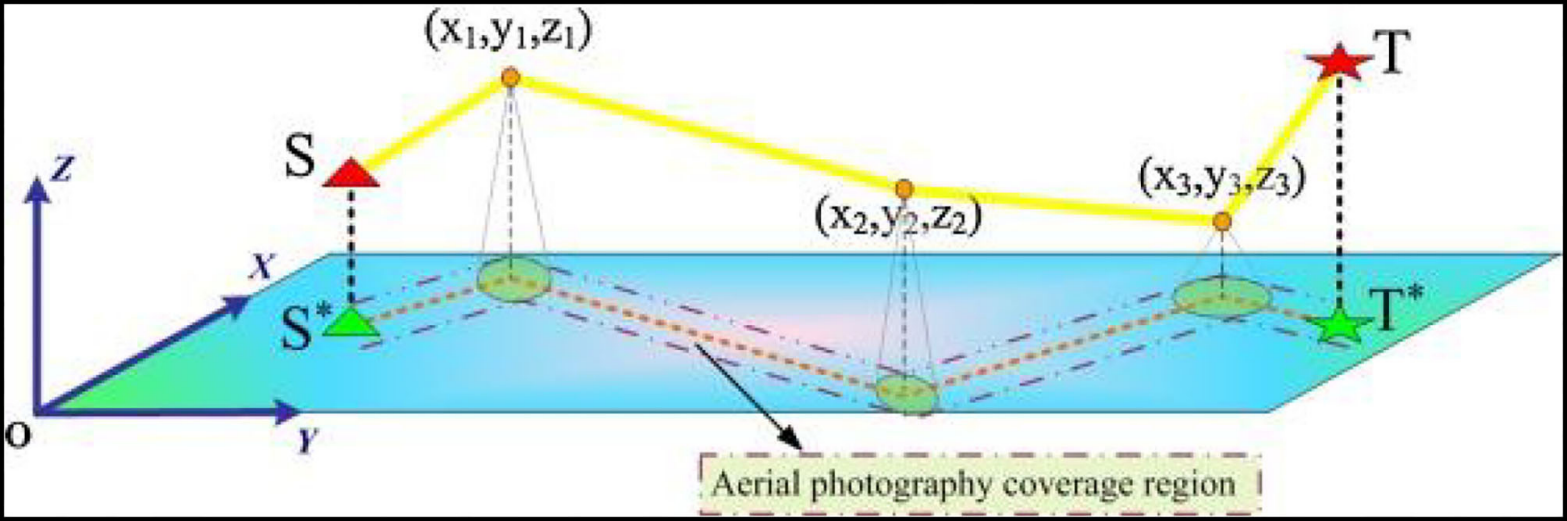
UAV flight route.

The main purpose of the UAV designed by this research institute is to identify the target with specific appearance and color. Therefore, the method of image processing and the calculation of the center of gravity of the target will be very important. This chapter will introduce how the UAV can recognize the appearance of the object. The steps of color processing and image processing are divided into: (1) color image capture, (2) RGB image grayscale, (3) median filtering, (4) image erosion and expansion, (5) binarization, (6) area filtering (7) shape discrimination (UAV flight design route shown in Figure 1).

#### Ground Control Platform System

The basic principle of digital image imaging is to collect energy by the photosensitive element, and normalize the energy to the range of the visible spectrum to produce “color.” A 2D digital image can be imagined as a function *f* (x, y). x, y represents the coordinate position, and *f* (x, y) is the color or grayscale value corresponding to the position. Digital images are sometimes interpreted as two-dimensional signals. Each point (x, y) in the image corresponds to the most basic element of the image; these elements are called “pixels.” Usually the grayscale of an image represents a set of grayscales in a single byte, or 8-bit. Therefore, there are 256 combinations representing 256 gray levels. The common color model is the RGB color model, which represents a color that can be divided into three red, green, and blue Channel. Each channel is represented by a byte, so a channel can represent 256 levels of color.

#### Aerial Photography System

The images captured in this experiment are color images in RGB format. For the color screening work, we take out the images of the RGB bins, then grayscale, and then judge the threshold of the binarization valve by the histogram, and finally the element binarized array is O-Red to segment the image of the target.

##### R, G, B Bit Surface Gray Scale

We will take a 640 × 480 color image taken by CCD, the content of which is a blue ceramic doll, take the R, G, B grayscale image of the image, and find the blue object in the B-bit grayscale image. Because the blue component is higher, the color is lighter. Because the blue and green components are lower than the R and G bins, the grayscale image is darker. We can use these differences to the image is split.

##### Gray Scale Distribution Analysis

We use gray scale histogram to analyze the gray scale distribution of the target in each element surface, and find the threshold range of object binarization. The highest peak is the most important distribution of background RGB gray scale value, and the second peak is doll. RGB grayscale value distribution.

##### Image Segmentation and Binarization

In order to track the target, we must segment the target image. Here we use the binarization of the image and the image operation to achieve the purpose of segmentation. In this study, we set a grayscale range, when the grayscale value of the image When it is within the range we set, make it black and set the value to 0. Otherwise, make it white and set the value to 1.

##### Median Filter

The image will generate noise during the binarization and segmentation process, so we use a median filter to eliminate these noises. The principle of the median filter is to place a mix-sized frame on each pixel, and list the gray-level or binarized data in the frame from small to large, in order to replace the original pixel corresponding to the frame. The grayscale value or binary value causes the noise to be eliminated.

##### Image Erosion and Expansion

Because we calculate the center of gravity of the target, we need a very complete image object, so we must mediate the target and then expand and erode (with a closed effect) to remove the voids and voids, in order to have a complete and no internal Cavity image to avoid misjudgment of coordinates of the center of gravity.

### System Software

The fuzzy controller designed in this study is the fuzzy control software built in MATLAB. The design steps are firstly to design the fuzzy set variables, and the attribution function to represent the degree of attribution of a parameter in the fuzzy set, and then according to the expert or the designer itself. Experience, each fuzzy set is made into a rule base, and then the calculation formula of the defuzzification is used to obtain the control quantity of the actual output of the fuzzy controller. Finally, the position and speed of the center of gravity of the target are treated as input variables, and the fuzzy controller and control output are connected to simulink blocks.

## Building Detection

### Experimental Design

The fuzzy controller designed in this study is based on the position of the target image captured by the CCD camera on the drone and the moving speed of the target on the image. It is blurred as the input variable of the paste controller, and I follow it. The research experience of the company sets its rule base, and the fuzzy inference engine determines the rules. Then, through the solution of the fuzzy interface, the angle of the front wheel needs to be rotated to achieve the purpose of tracking the target.

The function of fuzzification is to convert the explicit external input data into appropriate semantic fuzzy information, that is, to blur the explicit data into fuzzy information. In this paper, the position and moving speed of the target to be tracked are regarded as input variables. After defuzzification, an output variable is obtained for the front steering angle of the drone.

### Experimental Analysis

#### Data Source

**Figure 2:**
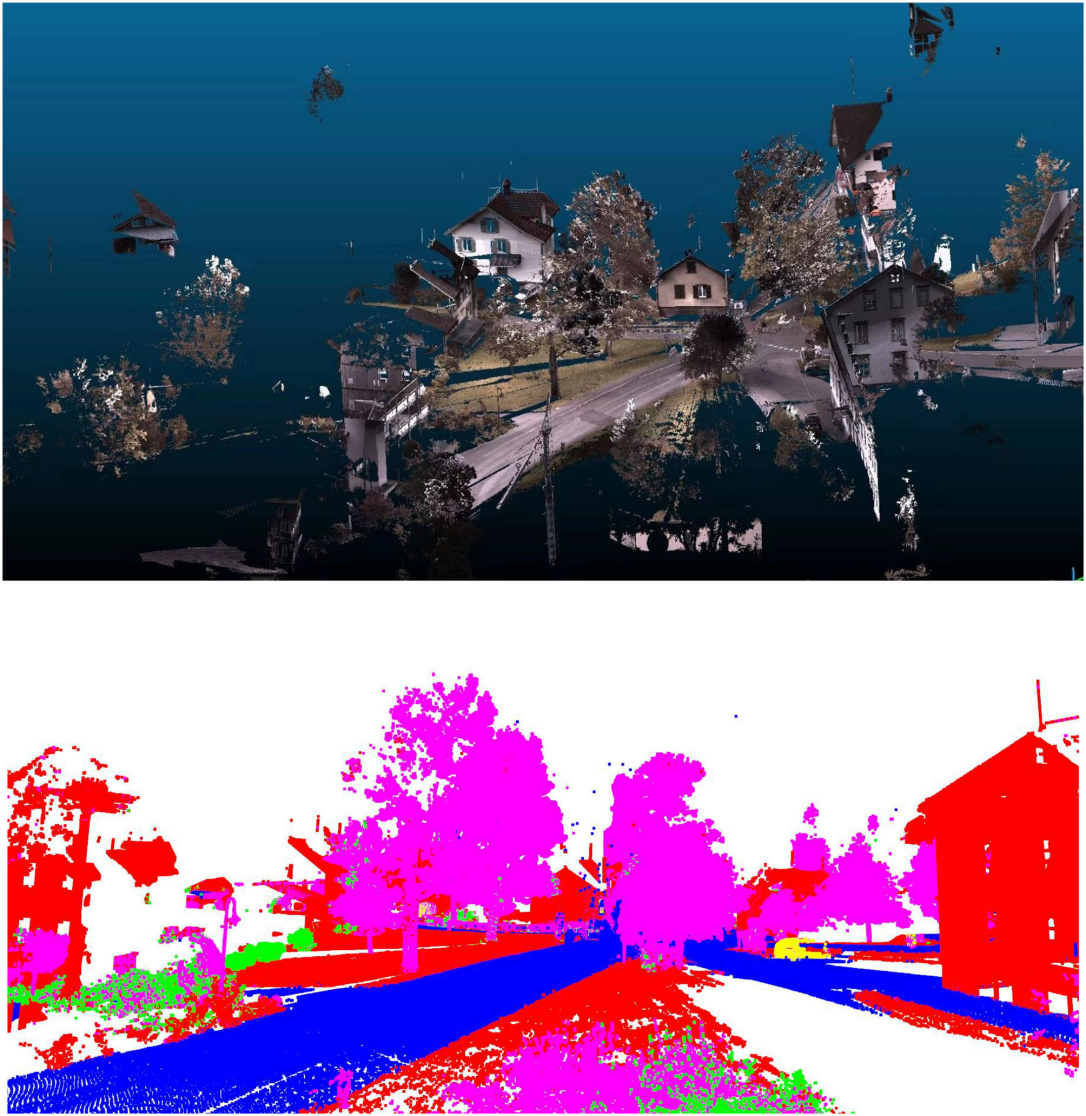
Object prediction recognition result.

When performing fuzzy control, the behavior of the controlled system is described by a set of fuzzy rules that use semantic fuzzy information rather than mathematical equations. Therefore, the knowledge of human experts can be transformed into fuzzy control rules. The fuzzy rule base is composed of multiple sets of If-Then type fuzzy rules to describe the input and output relationship of the system. This paper uses 25 sets of fuzzy control rules (results shown in Figure 2).

#### Analysis of Different Results

It can be seen from the results that, based on the characteristics of the path obstacles in the battlefield, this article stimulates the influence of the next target point on the ants, increases the positive feed back mechanism, and increases the probability of state transition. The improved ant colony algorithm improves the efficiency of path planning to a certain extent.

## UAV Path Optimization Design

### System Model

The main function of the wireless transmission subsystem is to transmit the images captured by the wireless CCD camera on the drone to the computer for image processing and application, and the computer also uses the wireless module to transmit control commands to the wireless drone to form a Control loop.

With the image transmission module, we can obtain the image information transmitted by the wireless CCD camera on the drone to the computer for subsequent processing and application. The image transmitter we selected here is a 2.4 GHz high frequency wireless 10 mW NTSC tubular camera. The receiver is a 2.4 GHz Wireless AV Sender Receiver PDMAVSR8000D.

Control command transmission module, its main function is to transmit the control output of the computer end after fuzzy operation to the BS2 microprocessor on the drone by wireless transmission, and control the rotation direction of the front wheel of the drone. The experiment uses a 27 MHz RF data transmission module, including a transmitting module and a receiving module, which can be used as the main components of PC wireless peripheral devices (such as wireless mouse, wireless keyboard) and various wireless remote controllers (such as remote control cars). Component. The main control computer and image processing subsystem is to take the image capture card device on the computer, and take the image captured by the CCD camera on the tracking car to make grayscale, binarize, calculate the invariant moment of the figure and obtain the image of the target object. The position of the center of gravity is then calculated by the fuzzy controller in MATLAB to calculate the amount of angular change that the front wheel should change, and finally transmitted to the BS2 single-chip controller subsystem to control the steering of the drone.

#### Model Description

##### Image Capture Card

The image capture card is selected from Dengchang Heng's UPG301B II image capture card.

* Use wafer: CONEXANT FUSION 878A.
* 32 bit PCI V2.1 specification, support plug and play interface (Plug and Play).
* Provides 2 sets of composite terminals (plum joints) and 1 set of S-VHS terminal inputs.
* Support NTSC/PAL video standards.
* Provides 320 × 240 BMP file single image capture function.
* Provides continuous motion image capture function of up to 320 × 240 AVI files.
* Support image brightness, contrast, chroma, chroma adjustment.
* Support Microsoft Media Encoder 9.0 for webcasting.
* Support Netmeeting video conferencing system.
* Provide SDK (Software Development Kit) software development library.

##### Chip Controller Subsystem

**Figure 3:**
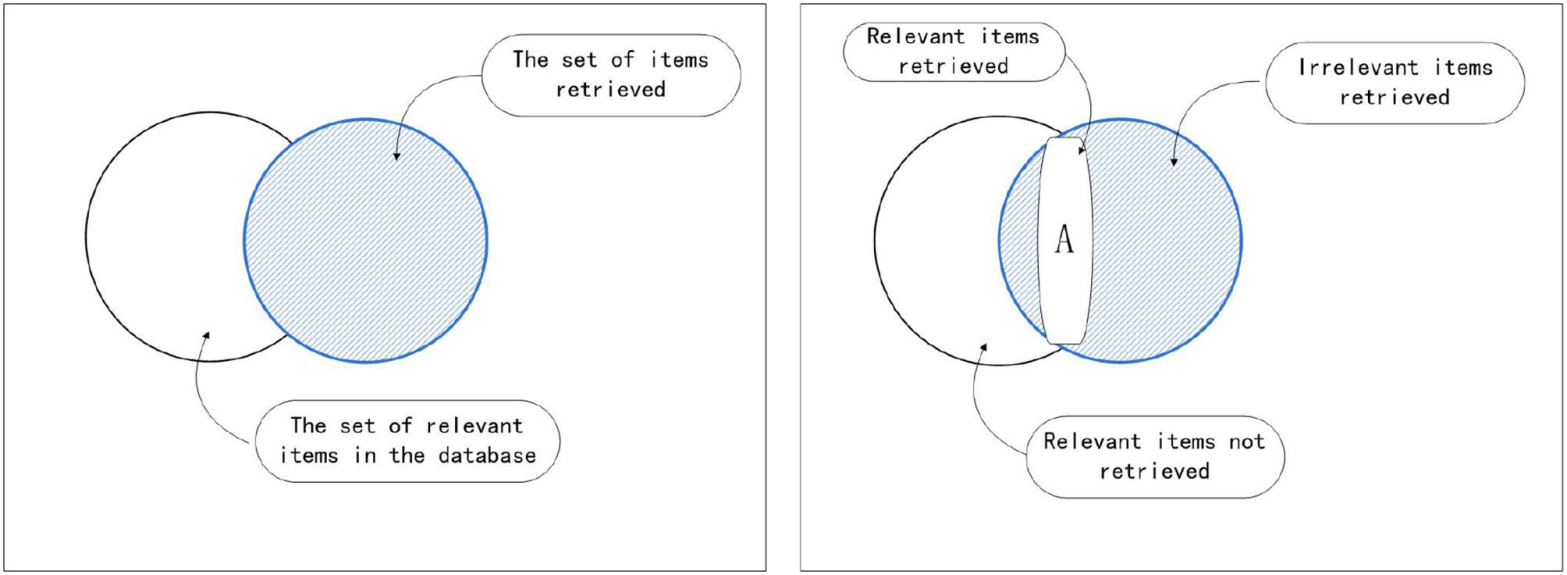
Recognition rate and recall rate explanation.

In this study, the BS2 microprocessor has two main functions. One is to convert the front wheel rotation control amount received by the wireless receiving module into a pulse wave signal for driving the stepping motor, so that the drone can move according to the command of the computer. The second is to drive the ultrasonic module to measure the distance of the target. When the distance between the drone and the target is <20 cm and the judgment tracking task is reached, the drone stops. This study used Parallax's BASIC Stamp 2, which has a microcontroller built on it. It is a black chip with the words - PIC16C57. Other parts used to make up BASI CStamp. All together, it has the correct name enbeded computer system. Also referred to as embedded systems (shown in Figure 3).

### Path Algorithm Design Introduction

The concept interpretation diagram obtained in this study is shown in the above figure. The experimental results show that there is a good discrimination degree, which is a more effective concept differentiation method.

According to this, the equation constructed in this study is:

(1) $$\bar{d_{ij}^{T_{1}}}=\alpha_{ij}^{T_{1}}d_{ij}^{T_{1}}\;当\;d_{ij}^{T_{1}}\leq \theta_{ij}^{T_{1}}$$

(2) $$\bar{d_{ij}^{T_{1}}}=\alpha_{ij}^{T_{1}}\theta_{ij}^{T_{1}}\;当\;d_{ij}^{T_{1}}>\theta_{ij}^{T_{1}}$$

(3) $$\bar{d_{ij}^{T_{2}}}=\alpha_{ij}^{T_{2}}(\bar{d_{ij}^{T_{1}}}-\theta_{ij}^{T_{1}}+d_{ij}^{T_{2}})\;当\;\bar{d_{ij}^{T_{1}}+}d_{ij}^{T_{2}}\leq \theta_{ij}^{T_{1}}+\theta_{ij}^{T_{2}}$$

(4) $$\bar{d_{ij}^{T_{2}}}=\alpha_{ij}^{T_{2}}\theta_{ij}^{T_{2}}\;当\;\bar{d_{ij}^{T_{1}}+}d_{ij}^{T_{2}}>\theta_{ij}^{T_{1}}+\theta_{ij}^{T_{2}}$$

(5) $$\bar{d_{ij}^{T_{3}}}=\alpha_{ij}^{T_{3}}(\bar{d_{ij}^{T_{2}}}-\theta_{ij}^{T_{2}}+d_{ij}^{T_{3}})\;当\;\bar{d_{ij}^{T_{\text{2}}}+}d_{ij}^{T_{\text{3}}}\leq \theta_{ij}^{T_{\text{2}}}+\theta_{ij}^{T_{\text{3}}}$$

(6) $$\bar{d_{ij}^{T_{\text{3}}}}=\;\alpha_{ij}^{T_{\text{3}}}\theta_{ij}^{T_{\text{3}}}\;当\;\bar{d_{ij}^{T_{\text{2}}}+}d_{ij}^{T_{\text{3}}}>\theta_{ij}^{T_{\text{2}}}\;+\;\theta_{ij}^{T_{\text{3}}}$$

Target building matrix in Matlab visualization:

(7) $$d^{T_{1}}=\left|\begin{array}{cccccccc} 0 & \;310 & \;2572 & \;1758 & \;1050 & \;1212 & \;2866 & \;4580\\ 0 & \;0 & \;1680 & \;845 & \;820 & \;930 & \;1500 & \;2060\\ 0 & \;0 & \;0 & \;750 & \;785 & \;766 & \;580 & \;2600\\ 0 & \;0 & \;0 & \;0 & \;720 & \;1011 & \;780 & \;2244\\ 0 & \;0 & \;0 & \;0 & \;0 & \;860 & \;1085 & \;1120\\ 0 & \;0 & \;0 & \;0 & \;0 & \;0 & \;1114 & \;2337\\ 0 & \;0 & \;0 & \;0 & \;0 & \;0 & \;0 & \;1280\\ 0 & \;0 & \;0 & \;0 & \;0 & \;0 & \;0 & \;0 \end{array}\right|$$

(8) $$d^{T_{2}}=\left|\begin{array}{cccccccc} 0 & \;560 & \;200 & \;430 & \;580 & \;600 & \;988 & \;1550\\ 0 & \;0 & \;225 & \;185 & \;602 & \;610 & \;698 & \;825\\ 0 & \;0 & \;0 & \;285 & \;298 & \;505 & \;666 & \;1000\\ 0 & \;0 & \;0 & \;0 & \;525 & \;596 & \;544 & \;1011\\ 0 & \;0 & \;0 & \;0 & \;0 & \;410 & \;526 & \;664\\ 0 & \;0 & \;0 & \;0 & \;0 & \;0 & \;394 & \;867\\ 0 & \;0 & \;0 & \;0 & \;0 & \;0 & \;0 & \;615\\ 0 & \;0 & \;0 & \;0 & \;0 & \;0 & \;0 & \;0 \end{array}\right|$$

(9) $$d^{T_{3}}=\left|\begin{array}{cccccccc} 0 & \;584 & \;360 & \;310 & \;230 & \;401 & \;288 & \;560\\ 0 & \;0 & \;648 & \;538 & \;720 & \;860 & \;449 & \;668\\ 0 & \;0 & \;0 & \;431 & \;160 & \;330 & \;240 & \;830\\ 0 & \;0 & \;0 & \;0 & \;424 & \;231 & \;244 & \;333\\ 0 & \;0 & \;0 & \;0 & \;0 & \;297 & \;363 & \;396\\ 0 & \;0 & \;0 & \;0 & \;0 & \;0 & \;391 & \;455\\ 0 & \;0 & \;0 & \;0 & \;0 & \;0 & \;0 & \;515\\ 0 & \;0 & \;0 & \;0 & \;0 & \;0 & \;0 & \;0 \end{array}\right|$$

**Figure 4:**
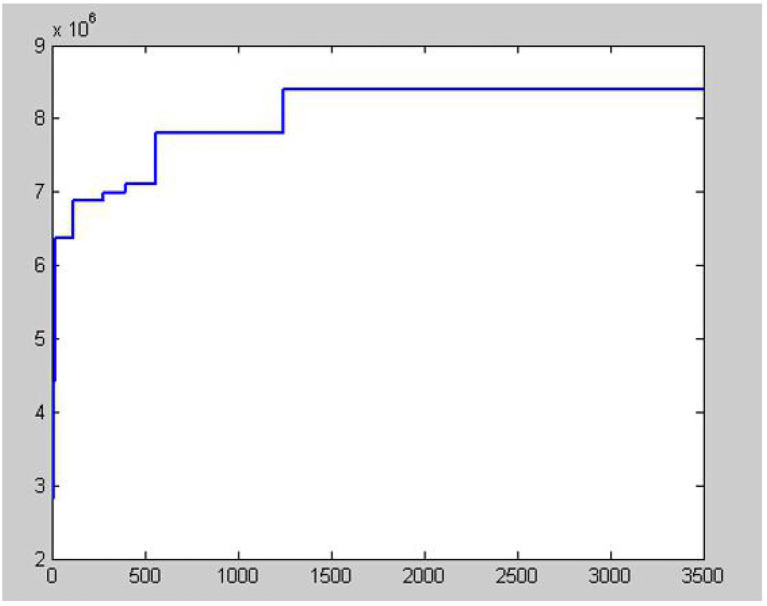
Algorithm convergence curve.

According to the above particle swarm and acoustic algorithm, the program solution is used, and the number of iterations is 3,500 times. The convergence curve is shown in the following figure. The calculation results are as follows (shown in Figure 4).

The target building iterative allocation matrix is:

(10) $$\theta^{T_{1}}=\left|\begin{array}{cccccccc} 0 & \;0 & \;2243 & \;0 & \;1239 & \;1109 & \;0 & \;4474\\ 0 & \;0 & \;0 & \;0 & \;0 & \;0 & \;0 & \;0\\ 0 & \;0 & \;0 & \;0 & \;814 & \;818 & \;0 & \;2780\\ 0 & \;0 & \;0 & \;0 & \;0 & \;0 & \;0 & \;0\\ 0 & \;0 & \;0 & \;0 & \;0 & \;969 & \;0 & \;1339\\ 0 & \;0 & \;0 & \;0 & \;0 & \;0 & \;0 & \;2701\\ 0 & \;0 & \;0 & \;0 & \;0 & \;0 & \;0 & \;0\\ 0 & \;0 & \;0 & \;0 & \;0 & \;0 & \;0 & \;0 \end{array}\right|$$

(11) $$\theta^{T_{2}}=\left|\begin{array}{cccccccc} 0 & \;0 & \;0 & \;370 & \;0 & \;714 & \;1053 & \;1744\\ 0 & \;0 & \;0 & \;0 & \;0 & \;0 & \;0 & \;0\\ 0 & \;0 & \;0 & \;0 & \;0 & \;0 & \;0 & \;0\\ 0 & \;0 & \;0 & \;0 & \;0 & \;651 & \;488 & \;1120\\ 0 & \;0 & \;0 & \;0 & \;0 & \;0 & \;0 & \;0\\ 0 & \;0 & \;0 & \;0 & \;0 & \;0 & \;352 & \;740\\ 0 & \;0 & \;0 & \;0 & \;0 & \;0 & \;0 & \;625\\ 0 & \;0 & \;0 & \;0 & \;0 & \;0 & \;0 & \;0 \end{array}\right|$$

(12) $$\theta^{T_{3}}=\left|\begin{array}{cccccccc} 0 & \;1454 & \;889 & \;2128 & \;810401 & \;3089 & \;560 & \;\;\\ 0 & \;0 & \;2553 & \;1568 & \;2142 & \;2400 & \;2647 & \;3553\\ 0 & \;0 & \;0 & \;1466 & \;458 & \;835 & \;1486 & \;1830\\ 0 & \;0 & \;0 & \;0 & \;1669 & \;1187 & \;1080 & \;2468\\ 0 & \;0 & \;0 & \;0 & \;0 & \;707 & \;1974 & \;1060\\ 0 & \;0 & \;0 & \;0 & \;0 & \;0 & \;1547 & \;582\\ 0 & \;0 & \;0 & \;0 & \;0 & \;0 & \;0 & \;1785\\ 0 & \;0 & \;0 & \;0 & \;0 & \;0 & \;0 & \;0 \end{array}\right|$$

As a new type of spatial data acquisition method, UAV low-altitude photogrammetry has the advantages of flexible operation, low cost and high resolution, and is widely used in many work areas. At present, in the selection of the route of overhead transmission lines, the 1:10,000 small-scale topographic map is generally used. Due to the obsolete topographic map information, it is difficult to select a scheme with less cross-streets, good geological conditions and short paths, and no one. The orthophoto acquired by the low-altitude photogrammetry technology has a unique advantage in the selection of the line path.

### Experimental Results Display

The document “2005–2030 UAV System Roadmap Planning” proposes that the US military's national security defense related departments have detailed descriptions of their military development plans for the next 25 years, considering the drones in the past and The current war and anti-terrorism have played a huge role. In the future, the unmanned aircraft cooperative warfare will still become an important mode of warfare. Especially in the collaborative path planning technology of task allocation, there will be rapid development. Accordingly, research on this issue will become a focus issue.

(1) Research on multi-target path planning for multi-UAV. Based on the characteristics of battlefield path obstacles, this paper aims to increase the positive feedback mechanism by stimulating the influence of the next target point on ants to increase the state transition probability. The initial pheromone intensity is inversely proportional to the target distance to adapt to no. The characteristics of evading threats during man-machine flight, improved ant colony algorithm to some extent improve the efficiency of path planning. However, in the battlefield environment, the path of the experimental simulation cannot completely replace the real path. The simulated path does not take into account the mechanical flight characteristics of the drone in actual flight, and does not smooth the path. Under the condition that all test parameters have been determined, the UAV faces the ever-changing battlefield space. Although the path planning of the two-dimensional plane has certain reference value, but there are limitations, then the path planning under the three-dimensional study will have Greater reference value and application value.
(2) Research on the assignment of multi-target multi-target tasks. In order to adapt to the dynamic target environment, this paper proposes a new heuristic task assignment algorithm based on the centralized algorithm and the general task assignment method. The algorithm introduces the path coincidence degree and adds the target point of the new path to the original planned path, achieving a certain degree of dynamic programming. However, the threshold values in the experimental data are set based on human experience and are not applicable to generality. And the algorithm is targeted when the drone performs other tasks such as providing fuel relay. How to improve the real-time allocation ability of the algorithm combined with the current hot research such as artificial intelligence, machine learning and big data analysis will also be the research direction of further expanding the algorithm.

## Conclusion

In summary, the drone designed by machine vision to distinguish contours is used to image the remote computer by wirelessly transmitting images. After several months, various MATLAB image processing programs have been developed. The purpose is to the target object is separated from the background environment, so that the computer can recognize the shape of the target object, and then calculate the position of the center of gravity of the target object and the speed of travel. After the fuzzy controller determines the optimal rotation angle, the computer automatically issues a wireless control command to make no Human function does not rely on human control, but can identify the target and track the moving target according to the fuzzy control method.

## Data Availability Statement

The raw data supporting the conclusions of this article will be made available by the authors, without undue reservation.

## Author Contributions

HZ was responsible for selecting the topic of the thesis, doing experiments, analyzing data, etc. and has approved this work for publication.

## Conflict of Interest

The author declares that the research was conducted in the absence of any commercial or financial relationships that could be construed as a potential conflict of interest.
